# Do Differences in Drinking Attitudes and Alcohol-Related Problems Explain Differences in Sick Leave? A Multilevel Analysis of 95 Work Units Within 14 Companies From the WIRUS Study

**DOI:** 10.3389/fpubh.2022.817726

**Published:** 2022-05-31

**Authors:** Neda S. Hashemi, Ingvild Dalen, Jens Christoffer Skogen, Hildegunn Sagvaag, David Gimeno Ruiz de Porras, Randi Wågø Aas

**Affiliations:** ^1^Department of Public Health, Faculty of Health Sciences, University of Stavanger, Stavanger, Norway; ^2^Department of Research, Section of Biostatistics, Stavanger University Hospital, Stavanger, Norway; ^3^Department of Quality and Health Technology, Faculty of Health Sciences, University of Stavanger, Stavanger, Norway; ^4^Department of Health Promotion, Norwegian Institute of Public Health, Bergen, Norway; ^5^Alcohol and Drug Research Western Norway, Stavanger University Hospital, Stavanger, Norway; ^6^Southwest Center for Occupational and Environmental Health, Department of Epidemiology, Human Genetics, and Environmental Sciences, School of Public Health in San Antonio, The University of Texas Health Science at Houston, San Antonio, TX, United States; ^7^Center for Research in Occupational Health (CiSAL), Universitat Pompeu Fabra, Barcelona, Spain; ^8^Consortium for Biomedical Research in Epidemiology and Public Health (CIBER en Epidemiología y Salud Pública-CIBERESP), Barcelona, Spain; ^9^Department of Occupational Therapy, Prosthetics and Orthotics, Faculty of Health Sciences, OsloMet – Oslo Metropolitan University, Oslo, Norway

**Keywords:** alcohol consumption, workforce, public health, attitudes, absenteeism, presenteeism

## Abstract

**Background:**

Systematic reviews have shown a strong relationship between alcohol consumption and sick leave. The effect of alcohol consumption on sick leave may, however, vary according to the work environment. While attitudes toward drinking may impact sick leave, there is little research on the contribution of drinking attitudes to sick leave. Moreover, alcohol-related problems and drinking attitudes may be influenced by the broader sociocultural contexts of the organizational units where people work.

**Objectives:**

This study aimed to explore the relationship of alcohol-related problems and drinking attitudes with sick leave while considering the nesting of employees within working units within companies.

**Method:**

Data from the WIRUS (Workplace Interventions preventing Risky alcohol Use and Sick leave) study were linked to company-registered sick leave data for 2,560 employees from 95 different work units in public (*n* = 9) and private companies (*n* = 5) in Norway. Three-level (employee, work unit, and company) negative binomial regression models were estimated to explore the 12-month prospective association of alcohol-related problems and drinking attitudes with four measures of sick leave (one-day, short-term, long-term, and overall sick leave days). Models were adjusted for gender, age, cohabitation status, educational attainment, work position, and employment sector.

**Results:**

We observed higher variation of one-day, short-term, and overall sick leave days between companies than between work units within companies (15, 12, and 30% vs. 0, 5, and 8%, respectively). However, neither alcohol-related problems nor drinking attitudes were associated with sick leave and, thus, those variations in sick leave were not explained by alcohol-related problems or drinking attitudes.

**Conclusion:**

Our findings suggest company-level differences are more important than within company differences when explaining differences in sick leave. While alcohol-related problems or drinking attitudes were not associated with sick leave, future studies may need to explore the role of company policies, practices, or social norms in variations in sick leave rates.

## Introduction

Health-related leaves have been linked to lifestyle behaviors, with alcohol consumption playing a major role (1–6). Risky alcohol use [i.e., a drinking pattern that raises the likelihood of medical, social, occupational, and economic problems (7)] increases the risk of long-standing illnesses and injuries (8–10) as well as mortality. For instance, in Europe, about 800 daily deaths are attributable to alcohol use and abuse (11). Alcohol consumption is not equal across Europe and, for instance, in 2018, one to three out of ten Norwegian employees were found to be characterized as risky drinkers (12). Norway's alcohol consumption (7.5 liters per capita in 2017) is higher than the average worldwide consumption of 6.4 liters per capita per year (13). Recent studies from Norway on alcohol and work impairment have found that alcohol consumption diminishes work performance (i.e., presentism) (3, 14). Risky alcohol use also can increase the risk of work-related injuries (15) and sick leave (16–18). One study on Norwegian employees found an increase of 13% in sick leave when the total alcohol consumption increased by one liter (19). Moreover, an Australian study reported that employees with monthly risky drinking patterns are about 8.7 times more likely to report alcohol-related sick leave than employees with low-risk drinking patterns (20).

The impact of alcohol consumption on sick leave could result in one or just a few days of absence due to alcohol intoxication and hangovers. For instance, employees are more likely to take a sick leave after consuming alcohol the previous night (21–23). The impact can also be related to long-term sick leave due to negative health and social effects of alcohol consumption over time (24, 25). However, the evidence on the relationship between alcohol consumption and sick leave is mixed. Several studies have found sick leave to be more likely to occur among individuals with alcohol-related problems (6, 19, 26–36), others report U-shaped associations (2, 24, 37, 38), and others have found no association (39–42) or negative associations (43), so that sick leave would be less common among those with higher levels of alcohol consumption. Some of the disparity in findings may be due to methodological differences in the operationalization of alcohol consumption and sick leave, or in the adjustment for confounders.

Sick leave imposes practical as well as financial burdens for individuals, businesses, and societies (5, 44, 45). Employees may face layoff consequences. Businesses may be forced to reschedule or reassign work duties to other existing employees or may need to recruiting temporary workers to mitigate the effect of a missing worker. The welfare system may need to absorb the cost of the leave (46–48). Sick leave, both in terms of spells and their duration, may be affected by a wide range of factors, including individual characteristics (e.g., age, gender), health conditions, working conditions, or the organization of work (49–51). Further, workers' decisions about their illness behavior may be affected by the ability to attend due to poor health but also by organizational values (52, 53). The workplace provides a significant cultural and social context in which, through social interaction processes, workers share and acquire knowledge regarding the expected behaviors and attitudes for effective participation in a work setting (54, 55). The interactions between characteristics of individuals and characteristics of working groups matter (56–58). Workgroup norms and attitudes toward drinking are found to be strong predictors of drinking behaviors (59–61) and work impairment (62). Moreover, workgroup norms concerning work attendance are suggested to be significant predictors of sick leave (63–65). Given this evidence, it is surprising that the majority of the prior research has focused mainly on the role of individual determinants. To fully understand the relationship between alcohol behavior and sick leave, it is important to assess the potential determinants at the individual (e.g., sociodemographic, drinking behaviors) and group levels (e.g., social norms and attitudes toward drinking). In addition, sick leave may also vary by business given differences in workplace's policies and practices regarding accruing and use of sick leave. Thus, there is a need to consider individual, group, and employer-level differences when studying the relationship between alcohol and sick leave.

Moreover, differences in sick leave also exist by country. These differences are related to variation in the definition of sick leave, culturally determined behaviors, and sick leave benefits schemes, which makes international comparisons challenging (66, 67). Even between Scandinavian countries, known by their similar approach to the welfare state (68), there are also differences, with Norway showing the highest rate of sick leave (46) before the COVID-19 pandemic started. During the COVID-19 pandemic, Norway still had the highest sick leave rate in the European Union (5.7%) (69, 70). Further, binge drinking is also frequent in Norway, which is a risk factor for short- and long-term health issues and social problems (11). The most recent study in Norway estimated that alcohol-related absence constitutes about 1% of the total sick leave and about 3% of short-term sick leave (71). However, no recent research has explored the relationship between drinking attitudes and sick leave in Norway.

Therefore, given the gaps identified in the literature, this study aimed to explore the relationship of alcohol-related problems and drinking attitudes with sick leave, while considering the nesting of employees within working units within companies in Norway.

## Materials and Methods

### Design

This study is part of the Norwegian national WIRUS (Workplace Intervention preventing Risky Use of Alcohol and Sick leave) project and was designed as a cohort study on a sample of employees in 14 companies in Norway. More details and other results from the WIRUS project are published elsewhere (3, 12, 14, 59, 72–80).

### Sample and Data Collection

Employees (blue, white, or pink-collar worker, or manager, i.e., a salaried worker) from 95 different work units were recruited from nine public and five private companies in Norway. These companies were categorized in accordance to the European Classification of Economic Activities (81), including: transportation and storage (*n* = 1), manufacturing (*n* = 3), public administration (*n* = 5), health care service (*n* = 3), accommodation (*n* = 1), and education (*n* = 1). The average work unit size had 27 employees (min. 10, max. 50).

A total of 17,855 employees from 19 companies were invited to participate in a web-based survey *via* their employer-provided e-mail addresses. Altogether, 5,076 employees accepted to complete the survey (28.5% response rate). WIRUS screening data regarding the included companies were collected from June 15, 2015 to 14 December, 2017. In 2020, company-registered sick leave data was collected for the 12-month follow-up period after each individual's baseline WIRUS screening (i.e., 2016 to 2018). Given delays due to the COVID-19 situation, data from five of the companies (*n* = 1,794 employees) was not available and, thus, these employees were excluded from the study. Further, after excluding participants without valid information on the key variables (e.g., alcohol-related problems, drinking attitudes, and sick leave), the final sample included 2,560 employees (50.4%) from 14 companies. Characteristics of the study sample are shown in Table 1.

**Table 1:** Study sample characteristics (*N* = 2,560).

**Characteristics**	**Study sample *n* (%)**
**Gender**	
Male	875 (34.2)
Female	1,685 (65.8)
**Age**	
≤39	780 (30.5)
≥40	1,780 (69.5)
**Cohabitation status**	
Living alone	357 (14.0)
Living with others	2,203 (86.0)
**Educational attainment**	
Primary/lower secondary	66 (2.6)
Upper secondary	568 (22.2)
University/college	1,926 (75.2)
**Work position**	
Worker^a^	2,062 (80.5)
Middle manager/senior executive	498 (19.5)
**Branches**	
Transport	62 (2.4)
Manufacturing	184 (7.2)
Public administration	1,647 (64.3)
Health care services	528 (20.6)
Accommodation	26 (1.0)
Education	113 (4.5)
**Employment sector**	
Private	275 (10.7)
Public	2,285 (89.3)

a *Including blue, white- and pink-collar workers*.

The final sample was predominantly female (*n* = 1,685; 65.8%), with more than two-thirds aged 40 or older, 14% reporting living alone, three out of four having completed university/college education, and approximately two out of ten being managers. Most respondents in the final sample (89.3%) were employed within the public sector companies (manufacturing, public administration, health care, and education), while the remaining were employed within private sector companies (transport, manufacturing, public administration, and health care). After comparing the study sample with the invited sample, only the proportion of employees age ≥40 was somewhat higher in the study sample (69.5 vs. 64.5%).

### Measures

#### Alcohol-Related Problems

The ten-item Norwegian translation of the Alcohol Use Disorders Identification Test (AUDIT) were used to measure alcohol-related problems. The AUDIT was developed by the World Health Organization (WHO) and is widely used to assess alcohol consumption and related problems in a wide range of settings and populations (7, 82). Each of the ten item is scored from 0 to 4, so the total score can range from 0 to 40. AUDIT covers three key domains including alcohol intake (items 1–3), dependence on alcohol (items 4–6), and alcohol-related harms (items 7–10). There is support for considering AUDIT as a one-factor tool indicating different levels of alcohol-related problems, as a two factor (drinking patterns and consequences) tool, or as three factors (drinking habits, alcohol dependence, and harmful alcohol use) (83, 84). However, the most recent confirmatory factor analysis of AUDIT based on WIRUS data (74) supports the use of AUDIT as a unidimensional measure of alcohol-related problems and so we used it as such in the present study. AUDIT's internal consistency in the present sample was acceptable (Cronbach's α = 0.78). For this study, we treated the AUDIT scores as a continuous sum score measure where higher scores indicate higher levels of alcohol-related problems.

#### Drinking Attitudes

Drinking attitudes were measured using the Norwegian translation of the Drinking Norms Scale (DNS) (85). The DNS is a 7-item scale addressing attitudes toward drinking in general (three items) and work-related drinking (four items). Earlier psychometric analyses have suggested using DNS as a unidimensional measure (85) and so we did in this study. Each item was coded on a 4-point Likert scale (1 = strongly disagree; 2 = disagree; 3 = agree; 4 = strongly agree). Negatively worded items (i.e., items 6 and 7) were reverse scored, and the total sum score for all seven items was calculated so that higher scores indicated more positive/liberal drinking attitudes. The DNS's internal consistency in the present sample was acceptable (Cronbach's α = 0.73).

#### Sick Leave

The primary outcome was the number of company-registered days of sick leave during the 12-month follow-up after the baseline WIRUS screening. Leaves due to maternity, pregnancy-related reasons, and non-health reasons (e.g., vacation) were excluded. We created three sick leave measures based on the total number of days on sick leave during the 12-month follow-up period (i.e., length of sick leave): short-term sick leave (i.e., ≤14 days, *n* = 1607, 62.7%, median: 5.0, IQR: 3.0–8.0), long-term sick leave (≥15 days, *n* = 348, 13.6%, median: 42.0, IQR: 21.0–89.0), and total sick leave as the total number of sick leave days within the 12 months of follow-up (*n* = 1632, 63.0%, median: 7.0, IQR: 3.0–25.0). In addition, for one-day sick leaves, the actual number of hours of sick leave taken within a day was registered. In Norway, the hours per week to which a full-time position equates is 37.5 and, so, a full-time working day would be 7.5 hours (86). Therefore, we created a ‘one-day sick leave hours' measure summing up the number of hours between 1 and 7.5 for all sick leaves which duration was no longer than 1 day (*n* = 1081, 42.0%, median: 11.0, IQR: 8.0–19.0).

For sensitivity analyses, we created additional metrics: for sick leaves of 14 days or less, we calculated an approximate number of days at risk (i.e., 365 minus total number of days of sick leave lasting longer than 14 days, assuming there could be a difference between a person who has only two short-term sick leaves (≤14 days) during the 12-month follow-up, and another who has several short-term sick leaves within 5 weeks but no long-term sick leave (≥15 days). In addition, we created four measures of sick leave spells [i.e., episodes (87)]: one-day hour (i.e., number of times a person had 1-day h sick leave, median: 2.0, IQR: 1.0–3.0), short-term spells (i.e., frequency of the short-term sick leave days, median: 3.0, IQR: 1.0–5.0), long-term spells (i.e., frequency of the long-term sick leave days, median: 2.0, IQR: 1.0–3.0), and total sick leave spells (i.e., frequency of having sick leave days of any durations, median: 3.0, IQR: 1.0–6.0).

### Covariates

Based on prior research (88–91), we included the following co-variables: gender (male, female), age (continuous), cohabitation status (living alone, living with others), educational attainment levels (primary/lower secondary, upper secondary, university/college), work position (employee, middle manager or senior executive), and employment sector (public, private).

### Analysis

Descriptive statistics are presented as frequencies and percentages for categorical variables, as means and standard deviations (SDs) for symmetrically distributed continuous variables, and as medians and interquartile ranges (IQRs) for asymmetric continuous variables.

Negative binomial (NB) regression models, crude and adjusted for gender, age, cohabitation status, educational attainment, work position, and employment sector, were used to assess the associations (incidence rate ratios or IRRs, with corresponding 95% confidence intervals or CIs) of alcohol-related problems and drinking attitudes with sick leave. Three-level random intercepts models were used to allow for intra-cluster correlation resulting from clustering of individuals within work units within companies. Sensitivity analyses were performed for short-term sick leave days by including the approximate number of days at risk as an exposure variable. In addition, the same analyses were performed for sick leave spells (87) to make sure that the results are consistent.

All descriptive analyses were performed using IBM SPSS, version 26. Multi-level regression models were running in Stata/SE version 17.0 (92), with function *menbreg*. Statistical significance was set at *p* < 0.05.

### Ethics

The study was approved to collect and store sensitive data by the Regional Committees for Medical and Health Research Ethics in Norway (REK) (approval no. 2014/647). Participants received an invitation letter and were informed about the overall aims of the WIRUS study and were assured that their participation was voluntary. All participants provided written informed consent prior to participation and were informed that they could withdraw their consent at any given time without any consequences. Respondents were treated according to the World Medical Association's Declaration of Helsinki (93).

## Results

The relationship of alcohol-related problems and attitudes with sick leave are shown in Table 2. Adjusting for gender, age [as a continuous variable], cohabitation status, educational attainment, work position and employment sector, alcohol-related problems showed no association with one-day (IRR = 1.00; 95% CI: 0.97–1.04), short-term (IRR = 0.99; 95% CI: 0.98–1.01), long-term (IRR = 0.96; 95% CI: 0.89–1.03), or overall sick leave days (IRR = 0.98; 95% CI: 0.95–1.00) on work units within companies. Similarly, drinking attitudes were not associated with one-day (IRR = 0.99; 95% CI: 0.96–1.04), short-term (IRR = 0.99; 95% CI: 0.96–1.01), and long-term days (IRR = 0.94; 95% CI: 0.88–1.01) on work units within companies. However, we found a slightly negative association between higher scores on drinking attitudes and taking sick leave (IRR = 0.97; 95 % CI: 0.95–0.99), indicating that one-unit higher score on drinking attitude was associated with 3% less sick leave days.

**Table 2:** Association^a^ of alcohol-related problems and attitudes with sick leave duration (one-day, short-term, long-term, and overall sick leave days), for 2,560 employees in 95 work units within 14 companies in the WIRUS study.

		**Sick leave**			
**Alcohol-related variables**		**One-day hours**	**Short-term days**	**Long-term days**	**Total days on sick leave**
Alcohol-related problems (continuous scores)^e^	IRR_crude_^b^	1.01	0.98	0.95	0.97 ^*^
	IRR_adjusted_^c^	1.00	0.99	0.96	0.98
	95% CI^d^	0.97−1.04	0.96−1.01	0.89−1.03	0.95−1.00
	Likelihood ratio *X^2^ p*-value	72.57 < 0.001	111.41 < 0.001	19.82 < 0.05	97.87 < 0.001
Drinking-attitudes (continuous scores)^f^	IRR_crude_^b^	0.99	0.98	0.94	0.96^**^
	IRR_adjusted_^c^	0.99	0.99	0.94	0.97
	95% CI^d^	0.96–1.02	0.96–1.01	0.88–1.01	0.95–0.99
	Likelihood ratio *X^2^ p*-value	72.64 < 0.001	111.90 < 0.001	20.34 < 0.05	99.76 < 0.001

a *Results from multilevel negative binomial regression analyses*;

b * IRR_crude_ = incidence rate ratio, bivariate association*;

c * IRR_adjusted_ = incidence rate ratio, adjusted association adjusted for gender, age, cohabitation status, educational attainment, work position, and employment sector*;

d * CI = confidence intervals*;

e * Composite score of the ten AUDIT items, potential range = 0–40, higher score indicates presence of alcohol-related problems*;

f * Composite score of the seven DNS items, higher score indicates positive/liberal drinking attitudes*;

* *p < 0.05*;

** *p < 0.01*.

The association between the covariates and sick leave is shown in Supplementary Table 1. Compared with males, females had higher one-day (IRR = 1.56; 95% CI: 1.27–1.92), short-term (IRR = 1.70; 95% CI: 1.44–2.00), long-term (IRR = 2.24; 95% CI: 1.61–3.11), and overall sick leave days (IRR = 1.66; 95% CI: 1.46–1.89) and age showed a slightly positive association with long-term days (IRR = 1.02; 95% CI: 1.01–1.03). Public sector employees had higher rate of taking sick leave than private sector employees and higher educational levels (i.e., upper secondary and university/college) was associated with less one-day, short-term, and overall sick leave days compared to lower educational levels (i.e., primary/lower secondary).

Sensitivity analysis showed that adjusting for days at risk did not affect the results noticeably (data not shown), nor did adjusting for age in two categories rather than continuously (shown in Table 1). Finally, using sick leave spells as the outcome measure rather than days did not alter the results (Supplementary Table 2).

All sick leave metrics showed statistically significant variation across companies, with short-term and overall sick leave also showing variation across work units within companies (Supplementary Table 3; Model 0). Between companies' variance in sick leave amounted to 15% of the total variance in one-day sick leave, and 12, 30, and 30% of the variance in short-term, long-term, and overall sick leave days, respectively. The variances in sick leave between work units within companies were generally lower. The co-variables (gender, age, cohabitation status, educational attainment, work position, and employment sector) explained much of the variation between companies, in particular for one-day and short-term sick leave (Model 1). The alcohol-related variables, on the other hand, explained little to none of the variation in sick leave (Models 2–3), and there were still substantial amounts of unexplained variation in long-term and overall sick leave days between companies in the fully adjusted model. The same results were obtained when adjusting for days at risk (data not shown).

## Discussion

This study aimed to explore the relationship of alcohol-related problems and drinking attitudes with sick leave, while considering the nesting of employees within working units within companies. The following main findings will be discussed: (i) most of the variance in sick leave (12–30% depending on the sick leave measure) was found between companies, while no more than 8% of the variance was found between work units within companies, (ii) alcohol-related problems showed no association with sick leave days, and (iii) drinking attitude showed no association with sick leave days, but showed a slightly negative association between higher scores on drinking attitudes and overall sick leave days between work units within companies.

The observed higher variation of sick leave between companies than between work-units within companies may be explained by differences in sick leave culture [i.e., self-awareness of others' or one's own attendance behavior or being agreed on a proper level of absence (94)] and social context, outside and inside the workplace (67, 95). Consistent with this notion, shared beliefs about absence and employment, and cultural salience (e.g., absence control system, existing technology, social ecology, friendship patterns, and communication) may be sensible reasons for variations in sick leave (95). For instance, compared to employees with higher empowerment in their jobs, employees having a lower sense of empowerment in their jobs have a stronger feeling of external control and, accordingly, have a concrete perception of taking sick leave (95, 96). However, organizational aspects such as colleagues' and supervisors' behaviors (1, 2, 97–99), the physical and mental load of the job (100, 101), workforce's downsizing (4), ethnic composition (102), job satisfaction (103), and psychiatric morbidity (104) may also contribute to the variation in sick leave between and within companies and their work units. Sick leave due to these factors can be considered as work-related sick leave and may have a greater need for being away from work than sick leave due to non-work-related factors (e.g., sick kids, flu) (105). Further, some of these factors may affect sick leave indirectly through the influence of health behaviors. For instance, colleagues' and supervisors' behaviors or job stress can influence a worker's consumption level of alcohol, which in turn may increase sick leave (1, 99).

Although several studies have explored the association of organizational culture and attitudes with sick leave (65, 106, 107), this study was the first to explore the association between drinking attitudes and sick leave. However, neither alcohol-related problems nor drinking attitudes explained sick leave in our study and drinking attitudes even showed a slightly negative association with overall sick leave. One may assume that companies characterized by more positive drinking attitudes can be characterized by more permissive absence norms, as they may take a more laissez-faire approach to control employees' behavior. However, as no association between alcohol-related problems and sick leave measures was found, finding no association between drinking attitudes and sick leave was unsurprising. In addition, as this study is the first to explore the association between drinking attitudes and sick leave, we thus cannot compare our observed results with other studies.

The lack of association between alcohol-related problems and sick leave is at odd with prior literature showing an alcohol-sick leave association, both among Norwegian employees (19, 22, 23, 29) and other populations (28, 30–36). However, our results are in agreement with other studies reporting no alcohol-sick leave association (39–42), including studies from Norway (39, 40). Overall, our study did not contribute to clarify the relationship between alcohol consumption and sick leave. Discrepancies in the literature may be attributed to several factors, mainly about differences in the measurement of exposure and outcome, the type of the organizations studied, or differences in study populations, which also make any direct national or international comparisons complicated.

Compared to other studies, different metrics of alcohol drinking levels and sick leave duration models were employed while referring to the same measure. For instance, in studies reporting an alcohol-sick leave association, short-term sick leave had been measured as ≤3 days (31), ≤7 days (32, 33), with self-reported measures (22, 29, 31), combined with other health issues as mental disorders or anxiety (30, 40). In some cases, there were differences in the measurement of alcohol consumption (e.g., average weekly volume, or alcohol use disorder) (28, 40). Moreover, although our results were consistent with some Norwegian studies (39, 40), those results were focused on individual-level factors and not company-level determinants.

Another reason for the existing discrepancy of findings regarding the association between alcohol-related individual differences and sick leave could be related to the work settings being studied. Some of the prior studies reporting an association between alcohol consumption and sick leave were using a sample of manual employees (19), non-industrial civil servants (32), police officers (34, 35), farm industry employees (36), or public sector employees (31). Although in the present study we used a sample from a wide variety of work settings, almost nine out of ten employees were employed within the public sector in a variety of occupations and industry settings. Some specific work settings may attract individuals with certain attitudes but, also, some shared attitudes and behaviors may form in such settings (65). Moreover, work settings reporting an alcohol-sick leave association may also be affected by the existing alcohol policies in place, birth cohort effect, social regulations, or alcohol availability at work.

Finally, the low participation rate in our study may have biased the associations toward the null. The healthy worker effect may have been compounded with the also known effect of non-responders in health surveys being generally less healthy than responders (108). People with drinking problems may be less prone to participate in surveys or to be in the workforce altogether (109) but also to provide inaccurate self-reports of alcohol consumption (110). Unfortunately, we were not able to control for any of these factors in our study, so more research would be needed to elucidate the true relationship of alcohol-problems and drinking attitudes with sick leave.

### Methodological Considerations

This study has several strengths. First, by using multilevel models, we were able to take into account the grouping of individuals within work units and companies. Second, we used company-registered sick leave data, which is considered a “gold standard” (111–114), one found to be valid and more reliable than self-reported sick leave data (46, 111, 115). However, there are some limitations to be considered when interpreting our results.

First, despite the large sample (*n* = 2,560), the study's response rate was low (14.3%), which may have implications for the representativeness of our study (116). Also, WIRUS study (3, 12, 59, 76) has an overrepresentation of females, employees with university/college education, employees age ≥40, and employees in the public sector compared with the overall Norwegian workforce. Studies state that health surveys have generally been skipped or underreported by (younger) men, individuals with lower socioeconomic status, and those having drinking problems (117–119), which may lead to an underestimation of the effect of alcohol on sick leave.

Second, alcohol-screening data was self-reported, which may have been affected by social desirability responses (SDR) as people tend to display a favorable image of themselves on surveys (120). However, SDR behavior does not undervalue employed validated and reliable alcohol measurement instruments (e.g., AUDIT). Another potential issue is recall bias. Shorter reference period may lead to more precise answers but reduce the ability to estimate one's typical alcohol consumption through a year (7, 121–123). Longer reference period (e.g., 1 year) are also recommended (121, 122) when using the AUDIT instrument.

Third, although the AUDIT is a -ecognize scale, other measures of alcohol such as the frequency or quantity of alcohol consumption may be related to sick leave. WIRUS did not included such measures so we could not compare our results with those studies measuring the amount of alcohol consumption.

Finally, although the results out of this study were adjusted for potential confounders, there might be other unmeasured factors of interest (e.g., mental health, diet, smoking, stress, or work conflict) (1, 24, 124, 125).

### Implications for Future Research

This study highlights the need for more refined measures and inclusion of other unmeasured factors to confirm the lack of associations of alcohol-related problems, drinking attitudes with sick leave. Also, one may clarify whether the existing high variation of sick leave between companies than between work units within companies is work-related or not. The attributable proportion of taking sick leave is reported to be higher for work-related sick leave factors than for lifestyle-related sick leave factors (124). Hence, knowing this difference may have significant implications not only for occupational risk prevention but also for the reduction of sick leave-related economic outcomes.

Moreover, more work is needed regarding the interaction between the type of employment, as well as the type of job position, and sick leave. Permanent employees tend to report more sick leave than non-permanent employees (49, 126), and employees in managerial positions report less sick leave, but more presentism, than other employees without such positions and responsibilities (127).

Therefore, further research is encouraged since the most recent study on the changes in alcohol consumption, among Norwegians, during the COVID-19 pandemic, has found a notable increase in proportion of heavy drinkers (128).

## Conclusions

Sick leave, which depends on multiple individual and contextual factors, is a key aspect of occupational health. Our study highlights the importance of between company-level differences over between work-units within company differences in relation to sick leave. The observed lack of associations between alcohol-related individual differences and sick leave suggests factors beyond individual characteristics such as organizational culture and the social context may play a role in the occurrence of sick leave. Hence, further research is needed to confirm or refute our findings in different settings while taking into consideration specific company policies or group norms.

## Data Availability Statement

The raw data supporting the conclusions of this article will be made available by the authors, without undue reservation.

## Ethics Statement

The studies involving human participants were reviewed and approved by the Regional Committees for Medical and Health Research Ethics in Norway (approval no. 2014/647). The patients/participants provided their written informed consent to participate in this study.

## Author Contributions

RA: conceptualization, project administration and funding acquisition. NH, ID, and RA: methodology and validation. NH and ID: formal analysis and data curation. NH: writing—original draft preparation. RA, JS, and HS: supervision. All authors: writing—review and editing. All authors have read and agreed, and supervision to the published version of the manuscript.

## Funding

The study was funded by the Norwegian Directorate of Health and the Research Council of Norway (Grant No. 260640). The funding bodies had no role in the design of study. DG was partly supported by the Southwest Center for Occupational and Environmental Health (SWCOEH), the Centers for Disease Control and Prevention (CDC) National Institute for Occupational Safety and Health (NIOSH) Education and Research Center (T42OH008421) at The University of Texas Health Science Center at Houston (UTHealth) School of Public Health: T42OH008421.

## Conflict of Interest

The authors declare that the research was conducted in the absence of any commercial or financial relationships that could be construed as a potential conflict of interest.

## Publisher's Note

All claims expressed in this article are solely those of the authors and do not necessarily represent those of their affiliated organizations, or those of the publisher, the editors and the reviewers. Any product that may be evaluated in this article, or claim that may be made by its manufacturer, is not guaranteed or endorsed by the publisher.
