# Supplementary material for: Do Differences in Drinking Attitudes and Alcohol-Related Problems Explain Differences in Sick Leave? A Multilevel Analysis of 95 Work Units Within 14 Companies From the WIRUS Study
Source: Front Public Health. 2022 May 31;10:817726. doi: 10.3389/fpubh.2022.817726 (PMC9194082; doi:10.3389/fpubh.2022.817726)
Supplement: Supplementary file 1 [file Data_Sheet_1.docx]

Supplementary Material

Supplementary Table 1. Association^a^ (IRR adjusted^b^ and 95% CI^c^) of sociodemographic and work-related characteristics with sick leave days for all 2,560 employees nested in 95 work units within 14 companies in the WIRUS study.

|  | | **Sick leave** | | | |
| --- | --- | --- | --- | --- | --- |
| **Characteristics** | | **One-day sick leave hours** | **Short-term sick leave** | **Long-term sick leave** | **Total days on sick leave** |
| **Gender** (Female vs. Male [Ref.]) | IRR_adjusted_ | 1.56 | 1.70 | 2.24 | 1.66 |
|  | 95 % CI | 1.27-1.92 | 1.44-2.00 | 1.61-3.11 | 1.46-1.89 |
| **Age** (in years) | IRR_adjusted_ | 0.98 | 1.00 | 1.02 | 0.99 |
|  | 95 % CI | 0.98-0.99 | 0.98-0.99 | 1.01-1.03 | 0.99-1.00 |
| **Cohabitation** **status** (Living with others vs. Living alone [Ref.]) | IRR_adjusted_ | 1.03 | 0.95 | 1.24 | 1.00 |
|  | 95 % CI | 0.78-1.36 | 0.81-1.11 | 0.84-1.83 | 0.86-1.18 |
| **Educational Attainment** (Upper secondary and University/college vs. Primary/lower secondary [Ref.]) | IRR_adjusted_ | 2.77 | 2.06 | 1.19 | 1.97 |
|  | 95 % CI | 1.47-5.21 | 1.44-2.93 | 0.49-2.89 | 1.37-2.83 |
|  | IRR_adjusted_ | 1.64 | 1.63 | 1.30 | 1.58 |
|  | 95 % CI | 1.22-2.20 | 1.39-1.91 | 0.88-1.93 | 1.35-1.86 |
|  | IRR_adjusted_ | 1.52 | 1.31 | 1.23 | 1.30 |
|  | 95 % CI | 1.21-1.91 | 1.15-1.50 | 0.88-1.72 | 1.14-1.48 |
| **Work** **position** (Middle manager and senior executive vs. Employee [Ref.]) | IRR_adjusted_ | 0.69 | 0.71 | 0.74 | 0.72 |
|  | 95 % CI | 0.53-0.91 | 0.60-0.83 | 0.50-1.10 | 0.61-0.84 |
|  | IRR_adjusted_ | 0.61 | 0.54 | 0.54 | 0.54 |
|  | 95 % CI | 0.33-1.16 | 0.36-0.80 | 0.19-1.50 | 0.36-0.80 |
| **Employment sector** (Public vs. Private employees [Ref.]) | IRR_adjusted_ | 0.62 | 0.62 | 0.61 | 0.62 |
|  | 95 % CI | 0.41-0.92 | 0.45-0.85 | 0.27-1.40 | 0.45-0.86 |
| Likelihood ratio *X^2^* | | 72.43  <.001 | 165.91  <.001 | 45.00  <.001 | 158.45  <.001 |
| ^a^ Results from multivariable (simultaneously adjusted for all the variables shown in the table) multilevel negative binomial regression analyses; ^b^ IRR_adjusted_ = incidence rate ratio, adjusted association; ^c^ CI = confidence intervals. | | | | | |

**Supplementary Table 2.** Association^a^ of alcohol-related problems and attitudes with sick leave (one-day, short-term, long-term, and overall sick leave spells) for 2,560 employees in 95 work units within 14 companies in the WIRUS study.

| **Alcohol-related variables** | | **Sick leave** | | | |  |
| --- | --- | --- | --- | --- | --- | --- |
|  |  | **One-day sick leave hours** | **Short-term sick leave** | **Long-term sick leave** | **Total days on sick leave** |  |
| **Alcohol-related problems (continuous scores)^e^** | IRR_crude_^b^ | 1.00 | 0.99 | 0.97 | 0.99 |  |
|  | IRR_adjusted_^c^ | 1.00 | 0.99 | 0.98 | 0.99 |  |
|  | 95% CI^d^ | 0.97-1.02 | 0.98-1.01 | 0.94-1.03 | 0.98-1.01 |  |
|  | Likelihood ratio *X^2^*  p-value | 143.63  <.001 | 165.90  <.001 | 45.45  <.001 | 157.56  <.001 |  |
| **Drinking-attitudes (continuous scores)^f^** | (IRR_crude_)^b^ | (0.99) | (0.98) | (0.96) | (0.98) |  |
|  | IRR_adjusted_^c^ | 0.99 | 0.98 | 0.97 | 0.98 |  |
|  | 95% CI^d^ | 0.97-1.01 | 0.97-1.00 | 0.93-1.01 | 0.97-1.00 |  |
|  | Likelihood ratio *X^2^*  p-value | 143.93  <.001 | 167.25  <.001 | 46.40  <.001 | 159.04  <.001 |  |
| ^a^ Results from multilevel negative binomial regression analyses; ^b^ IRR_crude_ = incidence rate ratio, bivariate association; ^c^ IRR_adjusted_ = incidence rate ratio, adjusted association adjusted for gender, age, cohabitation status, educational attainment, work position, and employment sector; ^d^ CI = confidence intervals; ^e^ Composite score of the ten AUDIT items, potential range = 0-40, higher score indicates presence of alcohol-related problems; ^f^ Composite score of the seven DNS items, higher score indicates positive/liberal drinking attitudes. | | | | | | |

**Supplementary Table 3.** Sick leave variation across companies and work units within companies (n=14) in the WIRUS study.

|  | RI variance (95% CI)  across companies | | | | RI variance (95% CI)  across work units within companies | | | |
| --- | --- | --- | --- | --- | --- | --- | --- | --- |
|  | One-day sick leave hours | Short-term sick leave | Long-term sick leave | Total days on sick leave | One-day sick leave hours | Short-term sick leave | Long-term sick leave | Total days on sick leave |
| Model 0^a^ | 0.15  (0.04-0.56) | 0.12  (0.04-0.40) | 0.30  (0.06-1.40) | 0.30  (0.11-0.83) | 0.00  (-----) | 0.05  (0.01-0.15) | 0.00  (-----) | 0.08  (0.03-0.22) |
| Model 1^b^ | 0.03  (0.00-0.64) | 0.04  (0.01-0.18) | 0.19  (0.03-1.26) | 0.19  (0.06-0.55) | 0.00  (-----) | 0.02  (0.00-0.17) | 0.00  (-----) | 0.07  (0.03-0.21) |
| Model 2a^c^ | 0.03  (0.00-0.62) | 0.04  (0.01-0.18) | 0.18  (0.03-1.27) | 0.19  (0.06-0.56) | 0.00  (-----) | 0.02  (0.00-0.17) | 0.00  (-----) | 0.07  (0.02-0.21) |
| Model 2b^d^ | 0.03  (0.00-0.69) | 0.04  (0.01-0.18) | 0.18  (0.02-1.27) | 0.18  (0.06-0.54) | 0.00  (-----) | 0.02  (0.00-0.17) | 0.00  (-----) | 0.07  (0.02-0.21) |
| Model 3^e^ | 0.03  (0.00-0.67) | 0.04  (0.01-0.18) | 0.17  (0.02-1.27) | 0.18  (0.06-0.54) | 0.00  (-----) | 0.02  (0.00-0.17) | 0.00  (-----) | 0.07  (0.02-0.21) |
| RI = Random Intercept; CI = Confidence Interval; ^a^ Empty model; ^b^ Adjusted for gender, age [continuous], cohabitation status, educational attainment, work position, and employment sector; ^c^ Adjusted for variables in Model 1 + alcohol-related problems; ^d^ Adjusted for variables in Model 1 +drinking attitudes; ^e^ Adjusted for variables in Model 1 + alcohol-related problems and drinking attitudes. | | | | | | | | |
